# Long-term electroacupuncture for low anterior resection syndrome in postoperative rectal cancer patients: case reports

**DOI:** 10.3389/fmed.2025.1517325

**Published:** 2025-06-18

**Authors:** Wenna Li, Ming Yang, Jinchang Huang, Qiaoli Zhang

**Affiliations:** ^1^The Third Affiliated Hospital, Beijing University of Chinese Medicine, Beijing, China; ^2^Institute of Acupuncture and Moxibustion in Cancer Care, Beijing University of Chinese Medicine, Beijing, China

**Keywords:** rectal cancer, low anterior resection, electroacupuncture, case report, Baliao points

## Abstract

This study reports two cases of rectal cancer patients who developed low anterior resection Syndrome (LARS) following rectal cancer surgery. Both patients presented with significant bowel dysfunction, including frequent defecation, urgency, fecal incontinence, and incomplete evacuation. Current treatments for LARS are limited by variable responses, high costs, and adherence issues, highlighting the need for practical, safe therapies with minimal side effects. The patients underwent a 6-month electroacupuncture treatment targeting Baliao points. Assessments were conducted using the LARS score, Wexner fecal incontinence score, and the EORTC QLQ-C30 scale. Results indicated a marked reduction in bowel frequency, significant relief of fecal incontinence symptoms, and improvement in overall health status and quality of life. In addition, emotional and cognitive functions were enhanced. These case reports suggest that electroacupuncture may be a valuable adjunctive treatment for managing LARS and improving patient emotional status and quality of life. Further high-quality research is necessary to evaluate the long-term efficacy of this treatment fully.

## 1 Introduction

Low Anterior Resection Syndrome (LARS) is a common complication after rectal cancer surgery, with a high incidence rate of up to 42–44% (1). After resection of rectal cancer, patients often present with LARS-related clinical symptoms, including frequent defecation, urgency, evacuation difficulties, and fecal incontinence (2). Low anterior resection syndrome is characterized as a functional disorder of the bowel following rectal resection, resulting in a decline in quality of life (3). Bowel dysfunction is associated with quality of life, especially for overall health status, social functioning, and role functioning (4, 5). Improving intestinal function and promoting overall well-being in postoperative patients with rectal cancer is a critical challenge.

Currently, there is still a lack of standardized and comprehensive treatment plans for LARS internationally (6). Interventions mainly include symptomatic treatment of defecation, pelvic floor rehabilitation, transanal irrigation, and sacral nerve stimulation therapy (7). Available treatment strategies have significant limitations, such as poor adherence and significant individual variability in treatment response, or high costs and the risk of infection (8). Recent researches highlight the development of new strategies for LARS management. The MANUEL project proposed evidence-based algorithms for LARS management, emphasizing personalized interventions (9). Therefore, a safe and effective therapy with few adverse effects is urgently needed (10).

Anal sphincter dysfunction and nerve injury are major causes of Low Anterior Resection Syndrome (LARS) in rectal cancer patients after surgery (11). Recent studies have indicated that traditional acupuncture may be a safe and effective approach to alleviate symptoms of LARS (12). The anatomical location of Baliao points corresponds to the posterior sacral foramina. The Shangliao (BL31), Ciliao (BL32), Zhongliao (BL33), and Xialiao (BL34) points are anatomically aligned with the first, second, third, and fourth posterior sacral foramina, respectively. The anterior and posterior sacral foramina are connected to the sacral canal, through which the anterior and posterior branches of the sacral nerves pass, respectively (13). The Baliao points are close to the sacral nerves, which innervate the muscles around the anus. Acupuncture at the Baliao points has been demonstrated to reduce anorectal pressure (14), improve pelvic floor nerve and muscle modulation, and promote recovery from defecation dysfunction (15). This could potentially help relieve symptoms in LARS patients, such as increased stool frequency and fecal incontinence (16). Electroacupuncture, an advancement of traditional acupuncture, enables controlled adjustment of intensity, frequency, and duration of stimulation, providing continuous stimulation, resulting in longer-lasting therapeutic effects (17). Electroacupuncture offers a promising option to clinical research on LARS, with advantages of high patient acceptance, minor trauma, and few adverse effects (18). This study presents two patients with rectal cancer showing severe LARS symptoms, including increased bowel frequency, fecal incontinence, urgency, and incomplete evacuation, demonstrating the potential of electroacupuncture treatment in improving symptoms of LARS, while also enhancing patients’ quality of life. This study was approved by the Institutional Review Board of the Third Hospital affiliated to Beijing University of Chinese Medicine (no. ECHBZYYSL-ZYDSY2024-13).

## 2 Study intervention and evaluation protocol

### 2.1 Electroacupuncture treatment protocol

**FIGURE 1:**
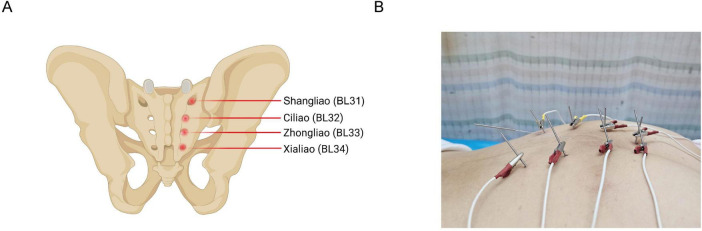
Anatomical locations of Baliao points and corresponding electroacupuncture needle placements. **(A)** Shows the anatomical locations of Baliao points, including Shangliao (BL31), Ciliao (BL32), Zhongliao (BL33), and Xialiao (BL34). **(B)** Shows positioning of electroacupuncture needles at Baliao points.

One well-trained person (T.A.) executed the electroacupuncture treatment. The patient was placed in a prone position, and the skin at the acupuncture points was disinfected. Disposable sterile acupuncture needles (0.30 × 75 mm) and an SDZ-V Hwato-brand electroacupuncture device were used during the treatment. The selected acupuncture points were Baliao points, including bilateral Shangliao (BL31), Ciliao (BL32), Zhongliao (BL33), and Xialiao (BL34) (Figure 1A). The needles were inserted in order from Xialiao to Shangliao. For Xialiao points, the needles were inserted vertically. For Zhongliao points, the needles were inserted obliquely at an approximate 70-degree angle to the skin, directed inferomedially. For Ciliao points, the insertions were at an approximate 50-degree angle, also directed inferomedially. Finally, for Shangliao points, the insertions were at an approximate 30-degree angle, pointing inferomedially. The patient experienced mild sensations of soreness, numbness, or distention, radiating toward the anus and perineum. Afterward, the SDZ-V electroacupuncture device was connected, with electrodes placed longitudinally, and a continuous wave with a frequency of 50 Hz was applied. The needles were retained for 30 min. Additionally, the Changqiang point (GV1) was obliquely needled for 30 min (Figure 1B). The 50 Hz continuous stimulation and 30-minute treatment duration have been used in electroacupuncture for postoperative gastrointestinal and bowel dysfunction, including rectal cancer patients (19, 20). These specific parameters have been demonstrated to improve bowel function improve bowel and enhance pelvic floor function (21). Based on our previous clinical experience and expert recommendations, these specific parameters may consistently improve bowel frequency, fecal incontinence, and overall quality of life. Our research team has applied the same electroacupuncture protocol targeting LARS in rectal cancer patients. This protocol has been executed in a single-arm trial, further supporting the efficacy of the parameters in our study (18). Furthermore, the acupuncture angles applied in our case reports are appropriate. The Baliao points are situated within the sacral foramina and must be inserted obliquely or perpendicularly to reach the sacral nerve branches. The acupuncture angles of the Baliao points can be adjusted based on clinical expertise (20, 22).

### 2.2 Clinical assessment tools

This study primarily utilized three scales to assess postoperative recovery in rectal cancer patients, including the LARS score (LARSS), the Wexner incontinence score, and the European Organization for Research and Treatment of Cancer Quality of Life Questionnaire Core 30 (EORTC QLQ-C30). These scales assessed defecation function, fecal incontinence, and overall quality of life.

LARSS is the only recognized tool globally used explicitly for assessing bowel function following LAR in rectal cancer patients. The total score ranges from 0 to 42, and is categorized into three severity levels, with 0–20 as no LARS, 21–29 as mild LARS, and 30–42 as severe LARS (23). We utilized the Chinese-translated and validated version, which has been previously adopted within the Chinese population and demonstrated high validity and reliability (24). The validated Chinese version has been widely used in research on bowel dysfunction following rectal cancer surgery.

The Wexner incontinence score offers a more comprehensive assessment of fecal incontinence. It evaluates symptoms related to incontinence for flatus, liquid stool, solid stool, wears pad usage and lifestyle alteration (25). This allows for a more detailed evaluation of changes in fecal incontinence symptoms (26). Wexner fecal incontinence score has been extensively applied in Chinese patient populations (27), including in postoperative patients of colorectal cancer, confirming its clinical feasibility and acceptability (28).

EORTC QLQ-C30 scale is a tool specifically designed for cancer patients that provides high specificity. It can evaluate multiple dimensions, including functional scales, symptom scales, and global health status, to comprehensively assess the quality of life in cancer patients (29). EORTC QLQ-C30 scale has been culturally adapted and psychometrically validated in China. It has demonstrated high reliability and validity (30, 31), and has been widely applied in clinical research among Chinese colorectal cancer patients (32).

## 3 Case presentation

### 3.1 Case no. 1

#### 3.1.1 Management of rectal carcinoma

A 66-year-old male patient was diagnosed with rectal adenocarcinoma with a TNM stage of pT3N2M0. The patient had no prior history of gastrointestinal disease, diabetes, or neurological disease and had not undergone abdominal surgery or pelvic intervention. He denied prior abdominal operations and did not have significant cardiovascular and endocrine comorbidities. In September 2023, the patient underwent abdominal computed tomography (CT) and colonoscopy due to increased bowel movement frequency and hematochezia. The examinations revealed a rectal mass 5 cm from the anal verge without distant metastasis. Pathological examination confirmed rectal tubular adenocarcinoma. A multidisciplinary team recommended neoadjuvant chemotherapy followed by surgery. The patient received two cycles of neoadjuvant chemotherapy with the capecitabine and oxaliplatin (CAPOX) regimen. The specific dosage included oxaliplatin 200 mg administered via intravenous infusion on day 1 of each cycle and capecitabine 1.5 g orally, twice daily from days 1 to 14, followed by a 7-day drug withdrawal. The patient received a total of two chemotherapy cycles. The patient completed neoadjuvant chemotherapy without any grade ≥ 3 toxicities, and the regimen was generally well tolerated. In November 2023, the patient underwent a sphincter-sparing proctectomy. The postoperative pathology revealed a poorly differentiated adenocarcinoma of the rectum (protruding type, size 6 × 6 × 3 cm). The tumor invaded the muscularis and extended into the perirectal adipose tissue, forming one cancerous nodule. Perineural invasion and intravascular emboli were noted. Metastasis was identified in 1 of 20 pericolic lymph nodes, and 5 of 10 no. 253 lymph nodes located at the root of the inferior mesenteric artery showed metastatic involvement. Immunohistochemistry results showed negative HER-2 expression (4B5: 0) and a high proliferative index (Ki-67 > 75%). The tumor exhibited positive staining for MLH1, MSH2, MSH6, and PMS2. From January to June 2024, the patient underwent six cycles of chemotherapy, with the regimen consisting of oxaliplatin 180 mg on day 1 and capecitabine 1.5 g orally, twice daily, from day 1 to day 14. The chemotherapy was well tolerated, with no significant adverse reactions observed. The patient did not undergo any interventions for LARS prior to electroacupuncture, including sacral nerve stimulation (SNS) or transanal irrigation.

#### 3.1.2 Electroacupuncture treatment and outcomes

The patient was severely affected by LARS symptoms after surgery, including frequent defecation, with up to 10 times a day, severely impacting his quality of life. In addition, frequent nocturnal bowel movements led to sleep disruption and considerable distress. Due to the persistence of LARS symptoms, the first patient, a 65-year-old male, began electroacupuncture treatment in February 2024, with a frequency of twice a week for a total of 6 months. The patients felt only minor discomfort from electrical stimulation. The discomfort was temporary and can be alleviated by modifying the electrical current. During the first 4 weeks after rectal cancer surgery, the patient followed a low-fiber, low-fat, and easily digestible soft diet, with adequate fluid intake. After six weeks, the diet gradually returned to normal, while caffeine, alcohol, and spicy foods were avoided. These measures followed the routine postoperative management of rectal cancer and did not constitute structured interventions targeting LARS. No other LARS-related interventions were implemented during the treatment period, including structured dietary or lifestyle interventions, PFR, TAI, or SNS. Therefore, the clinical improvements observed in both cases are more likely attributable to electroacupuncture alone.

**FIGURE 2:**
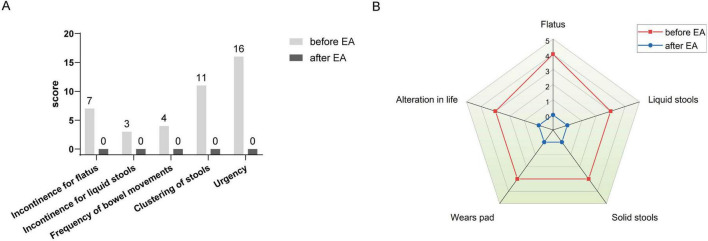
LARSS and Wexner incontinence score before and after electroacupuncture treatment in the first patient. **(A)** Shows LARSS scores of incontinence for flatus and liquid stools, frequency of bowel movements, clustering of stools, and urgency before and after treatment. **(B)** Shows Wexner incontinence scores before and after treatment, including incontinence for flatus, liquid stool, and solid stool, as well as wears pad usage and lifestyle alteration. EA, electroacupuncture.

After rectal cancer surgery, the patient experienced more than ten bowel movements per day, accompanied by urgency and incomplete evacuation, with a LARSS score of 41. During the initial 3 months of electroacupuncture treatment, the frequency of bowel movements decreased from 10 times per day to approximately 5 times. Night-time bowel movements also decreased, allowing the patient to sleep better. By the end of the 6-month treatment, the symptoms of fecal incontinence, urgency and incomplete evacuation had improved significantly, with bowel frequency stabilizing 1 to 3 times per day, which was manageable for the patient. The patient’s LARSS rating was assessed after 6 months of electroacupuncture treatment, and it improved from severe LARS (41 points) to no LARS (0 points) (Figure 2A). The Wexner score also improved, dropping from 17 before treatment to 0 (Figure 2B). In addition, according to the EORTC QLQ-C30 scale, we found that electroacupuncture treatment led to significant improvements in functional dimensions, including physical, emotional, cognitive, and social functions, with post-treatment scores increasing to 100. Symptoms such as fatigue, pain, and nausea were completely alleviated, with scores dropping to 0. Furthermore, sleep quality improved, and anxiety and depressive feelings related to bowel urgency were reduced. The patient’s overall health and quality of life were significantly enhanced (Table 1). Follow-up in February 2025 showed that the patient had 1–2 bowel movements per day. There were no bowel movements at night, and sleep was not disturbed. Stool appearance was normal, and bowel movements were regular. There was no more fecal incontinence or urgency. Both the LARSS and Wexner scores were 0, signifying the resolution of intestinal dysfunction. The EORTC QLQ-C30 questionnaire indicated that the patient sustained a positive status in all dimensions, including physical, emotional, cognitive and social functioning. The global health score increased from 75 after treatment to 83. Symptoms like fatigue and pain did not come back. Sleep quality stayed good. The patient felt emotionally stable and reported a better quality of life. Case no. 1 outcomes at different time points are shown in Table 2.

**TABLE 1:** EORTC QLQ-C30 scores before and after electroacupuncture treatment for the first patient.

Dimension	Item numbers	Pre-treatment score	Post-treatment score
**Functioning subscale**			
Physical functioning	Q1–Q5	27	100
Role functioning	Q6–Q7	33	100
Emotional functioning	Q21–Q24	33	100
Cognitive functioning	Q20, Q25	33	100
Social functioning	Q26–Q27	83	100
**Symptom subscale**			
Fatigue	Q10, Q12, Q18	88	0
Nausea and vomiting	Q14, Q15	50	0
Pain	Q9, Q19	83	0
Dyspnea	Q8	100	0
Insomnia	Q11	100	0
Appetite loss	Q13	100	0
Constipation	Q16	67	0
Diarrhea	Q17	100	0
Financial difficulties	Q28	0	0
**Global health status subscale**			
Global health status	Q29–Q30	17	75

**TABLE 2:** Case no. 1: patient bowel function and symptoms at different time points.

Time points	Bowel frequency*	Fecal incontinence*	Fecal urgency*	Sleep quality	Emotional state	LARSS score	Wexner score	EORTC QLQ-C30 (Global Health Score)
Before EA (Feb 2024)	10–15 times/day	1–3 times/day	3–5 times/day	Poor	Anxiety/ depression	41	17	17
After EA (Aug 2024)	1–3 times/day	None	None	Good	Positive	0	0	75
Follow-up (Feb 2025)	1–2 times/day	None	None	Good	Positive	0	0	83

*Bowel Frequency, The daily frequency of bowel movements, including both daytime and nocturnal occurrences; Fecal Incontinence, The frequency of involuntary expulsion of a large volume of fecal material; Fecal Urgency, The frequency when the patient experiences an immediate compulsion to defecate, accompanied by an inability to postpone bowel movement.

### 3.2 Case no. 2

#### 3.2.1 Management of rectal carcinoma

A 65-year-old male patient underwent a colonoscopy in April 2022 due to bloody stools and perianal pain. The colonoscopy showed a rectal mass located 6 cm from the anal verge, and pathological examination confirmed rectal tubular adenocarcinoma. Contrast-enhanced abdominal CT and pelvic MRI revealed a mid-rectal tumor extending beyond the muscularis propria, with no signs of regional lymphadenopathy or distant metastases. The patient had no prior history of gastrointestinal, neurological, cardiovascular, or endocrine disorders. He had not undergone any previous abdominal or pelvic surgery. The patient underwent proctectomy in May 2022. The postoperative pathology revealed a moderately differentiated adenocarcinoma of the rectum (protruding type, size 5.5 × 5 × 2.5 cm). The tumor invaded the muscularis and extended into the perirectal adipose tissue. Perineural invasion was noted, but no evidence of vascular invasion was detected. No regional lymph node metastasis was observed. Immunohistochemistry results showed negative HER-2 expression (4B5: 0) and a moderate proliferative index (Ki-67: 75%). The tumor exhibited positive staining for MLH1, MSH2, MSH6, and PMS2 with a TNM stage of pT3N0M0. After surgery, the patient received eight cycles of a capecitabine chemotherapy regimen. The specific dose was 1.25 g, orally twice daily from days 1 to 14 (d1–d14) of each chemotherapy cycle, followed by a 7-day drug withdrawal. Each cycle lasted 21 days, and the patient completed eight cycles. During chemotherapy, the patient experienced nausea and fatigue. No grade ≥ 3 hematologic or gastrointestinal toxicities were observed. The patient did not undergo any LARS intervention postoperatively, including SNS or transanal irrigation.

#### 3.2.2 Electroacupuncture treatment and outcomes

The patient’s primary postoperative symptoms included an increased frequency of defecation, up to 20 times a day, which was difficult to control, accompanied by anal pain and discomfort, as well as periodic intestinal fluid leakage. The patient had generalized weakness, affecting daily life seriously. The patient underwent 6 months of electroacupuncture therapy at our hospital 1 year after the operation, with treatments administered twice per week. The electroacupuncture protocol was the same as that used for the first patient. Following 30 min of stimulation, the patient felt mild skin irritation at the needling sites. The current intensity (1–5 mA) was adjusted to induce slight skin twitching around the acupoint without pain. Additionally, each treatment session was limited to 30 min to minimize the risk of side effects caused by extended electrical stimulation. The patient followed general postoperative recommendations after rectal cancer surgery, including consuming a light diet, avoiding irritant foods, and maintaining a regular daily routine. During the treatment period, no additional interventions for LARS were implemented, such as structured dietary or lifestyle interventions, PFR, TAI, or SNS.

**FIGURE 3:**
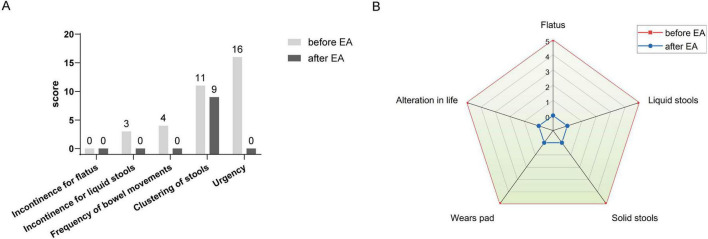
LARSS and Wexner incontinence score before and after electroacupuncture treatment in the second patient. **(A)** Shows scores of incontinence for flatus and liquid stools, frequency of bowel movements, clustering of stools, and urgency before and after treatment. **(B)** Wexner incontinence scores before and after treatment, including incontinence for flatus, liquid stool, and solid stool, as well as wears pad usage and lifestyle alteration. EA, electroacupuncture.

Following the rectal cancer surgery, the patient had significant bowel dysfunction, with more than twenty bowel movements per day, accompanied by a feeling of anal discomfort. The LARSS score was 34, indicating severe LARS. The patient’s symptoms gradually improved during the first 3 months of electroacupuncture treatment. The daily frequency of bowel movements gradually decreased from over twenty to about 10, while the comfortlessness and pain diminished, and urgency was significantly reduced. The patient’s overall emotional state improved, which further enhanced the patients confidence in the treatment and provided a strong foundation for continued care. After 6 months of electroacupuncture treatment, a reassessment of the patient’s LARSS score showed a reduction from severe LARS (34 points) to mild LARS (9 points), indicating that the patient’s bowel function had nearly returned to normal (Figure 3A). Bowel frequency stabilized at 2–3 times per day, with the sensation of anal discomfort and pain nearly disappearing, and complete restoration of strength. The Wexner scale score decreased from 20 before treatment to 0 (Figure 3B). The patient’s ability to control bowel movements has improved significantly, and fecal incontinence has completely disappeared, which has dramatically improved his quality of life. The results of the EORTC QLQ-C30 scale showed that prior to treatment, the patient had severe dysfunction in multiple dimensions, especially fatigue, pain, and diarrhea, which seriously affected daily life and overall health. Following electroacupuncture treatment, the patient’s physical function recovered significantly, scoring 93. Role function improved to 67, emotional function improved to 83, cognitive function score improved to 50, and social function remained at 50. Most notably, the symptoms of fatigue, pain, and diarrhea completely alleviated, with scores dropping to 0. The patient’s overall health status improved significantly, with the global health score rising from 0 to 100, indicating a substantial improvement in quality of life and health following treatment (Table 3). At the November 2024 follow-up, the patient maintained a bowel movement frequency of 2–3 times per day and regular bowel movements, with no incontinence or urgency. The LARSS score remained at 9 points, and the Wexner score remained at 0. According to the EORTC QLQ-C30, the patient’s physical function remained good, emotional status was positive, and the global health score remained 100 points. The patient’s quality of life was not affected by intestinal issues, with overall health status. Case no. 2 outcomes at different time points are shown in Table 4.

**TABLE 3:** EORTC QLQ-C30 scores before and after electroacupuncture treatment for the second patient.

Dimension	Item numbers	Pre-treatment score	Post-treatment score
**Functioning subscale**			
Physical functioning	Q1–Q5	20	93
Role functioning	Q6–Q7	33	67
Emotional functioning	Q21–Q24	25	83
Cognitive functioning	Q20, Q25	17	50
Social functioning	Q26–Q27	50	50
**Symptom subscale**			
Fatigue	Q10, Q12, Q18	100	0
Nausea and vomiting	Q14, Q15	0	0
Pain	Q9, Q19	100	0
Dyspnea	Q8	0	0
Insomnia	Q11	0	0
Appetite loss	Q13	0	0
Constipation	Q16	0	0
Diarrhea	Q17	100	0
Financial difficulties	Q28	0	0
**Global health status subscale**			
Global health status	Q29–Q30	0	100

**TABLE 4:** Case no. 2: patient bowel function and symptoms at different time points.

Time points	Bowel frequency*	Fecal incontinence*	Fecal urgency*	Sleep quality	Emotional state	LARSS score	Wexner score	EORTC QLQ-C30 (Global Health Score)
Before EA (May 2023)	20–30 times/day	3–5 times/day	4–5 times/day	Fair	Anxiety/ depression	34	20	0
After EA (Nov 2023)	2–3 times/day	None	<1 time/week	Good	Positive	9	0	100
Follow-up (Nov 2024)	2–3 times/day	None	<1 time/week	Good	Positive	9	0	100

*Bowel Frequency, The daily frequency of bowel movements, including both daytime and nocturnal occurrences; Fecal Incontinence, The frequency of involuntary expulsion of a large volume of fecal material; Fecal Urgency, The frequency when the patient experiences an immediate compulsion to defecate, accompanied by an inability to postpone bowel movement.

## 4 Discussion

Electroacupuncture is generally considered a safe treatment in rectal cancer patients (33). In this study, no needling-related adverse reactions were observed in all subjects. Patients were closely monitored throughout the treatment period, particularly during the initial electroacupuncture session, to prevent potential adverse reactions. The findings revealed that side effects from electroacupuncture on Baliao points were mild and temporary, consistent with the safety profiles observed in previous studies (14). Compared to conventional interventions, electroacupuncture offers a minimally invasive approach with fewer associated risks. Sacral nerve stimulation (SNS) involves the surgical implantation of a device to stimulate the S3 sacral nerve. This process carries risks such as infection, device failure, and the need for ongoing maintenance (34). Electroacupuncture is non-invasive and does not involve surgery, hence eliminating these risks. Furthermore, electroacupuncture shows distinct advantages in patient adherence. SNS requires permanent implantation and intricate parameter adjustments (35), while electroacupuncture is non-invasive and allows adaptable stimulation according to patient tolerance (36). Transanal irrigation demands daily use, frequently resulting in suboptimal compliance due to pain and discomfort (37). Conversely, electroacupuncture causes minimal interference with daily life and is better accepted. Pelvic floor rehabilitation requires active participation and prolonged training, restricting its use in patients with severe symptoms (38). Electroacupuncture works via neuromodulation and does not depend on patient effort, offering broader accessibility.

In addition to its positive safety and good adherence, electroacupuncture may ameliorate nerve injury and alleviate frequent defecation symptoms in postoperative LARS by sustained neural stimulation (16). Intraoperative damage to the sympathetic and parasympathetic nerves is a key factor contributing to postoperative LARS in patients with rectal cancer (39). Low anterior resection for rectal cancer leads to loss of autonomous control of the anal sphincter and incoordination of the rectal smooth muscles with the pelvic floor muscles (40). Pelvic floor rehabilitation effectively improved bowel control by strengthening the pelvic muscles (41). In contrast, acupuncture is recognized as a neuromodulatory therapy that activates somatic sensory afferents and modulates autonomic and neurochemical pathways involved in visceral regulation (42). It exerts broader neuromodulatory effects, potentially leading to faster and more pronounced robust improvements in the bowel (43). Colonic motility may be altered through the modulation of enteric nervous system with acupuncture, lowering urgency symptoms of LARS (44).

Changqiang (GV1), located near the coccyx, is richly innervated. The deeper layers are penetrated by branches of the pudendal, perineal, and inferior rectal nerves. Stimulation of the GV1 via acupuncture will enhance coordination between reflexes of the pelvic floor and rectal sensation to improve bowel control and minimize fecal incontinence (45). Shangliao (BL31) is at the origin of the sacrospinalis and gluteus maximus muscles, under which the first sacral nerve (S1) passes. Ciliao (BL32) and Zhongliao (BL33) are at the gluteus maximus origin, and are passed through the posterior branches of the second (S2) and third (S3) sacral nerves, respectively. Xialiao (BL34) is at the similar muscular origin, with the fourth sacral nerve (S4) lying along with it (46). Baliao points are close to the sacral plexus, which innervates the pelvic floor muscles, including the levator ani, the external anal sphincter, and the internal anal sphincter through autonomic fibers (47). Deep needling of these points will directly stimulate the sacral nerve roots and potentially modulate both the somatic and the autonomic pathways used in anorectal function (20). Further, stimulation of the nerve via these points could increase the contractility of the external anal sphincter and thus enhance fecal continence control (48). Electroacupuncture has some advantages over traditional acupuncture. Electroacupuncture provides rhythmic microcurrent stimulation to the rectal surrounding muscles, inducing rhythmic contraction and enhancing the coordination of the muscles (21). Electroacupuncture at the Baliao acupoint was found to activate the sacral nerves (49), thus ensuring the regulation of the function of defecation (50). Sacral nerve stimulation (SNS) mainly treats fecal incontinence by implanting electrodes into the sacral foramina and stimulating the sacral nerves using low-frequency pulses (51, 52). SNS typically targets only the S3 nerve. In comparison, electroacupuncture at Baliao points stimulates the S1-S4 nerves, modulating defecation function via the sacral nerves. Therefore, in this study, electroacupuncture at Baliao points was used as the primary intervention to explore its efficacy in treating LARS.

Although the initiation time of electroacupuncture treatment differed, with Case 1 initiating at 3 months and case 2 initiating at 12 months after rectal cancer surgery. Both of them demonstrated improvement in bowel function. It has been shown that spontaneous improvement in symptoms of LARS may occur within the first 12 months after rectal cancer surgery (53). However, this spontaneous improvement does not exclude a potential therapeutic effect of electroacupuncture. In case no. 2, electroacupuncture commenced 12 months after surgery, the patient’s symptoms have stabilized. Therefore, the subsequent improvement observed may be more directly attributed to the effects of electroacupuncture. After intervention, the LARS score decreased from severe to mild, accompanied by a reduction in bowel movement frequency and urgency. These observations imply that delayed electroacupuncture can have potential therapeutic benefits in LARS patients. Indeed, electroacupuncture restores bowel function instead of relying on spontaneous recovery (54, 55). As stated above, electroacupuncture at the points of Baliao can stimulate the sacral roots of the nerves (S1–S4), thereby controlling the anorectal muscles and the pelvic floor. The ongoing neural stimulation enhances the neuromuscular coordination and gradually enhances the function of defecation. The extensive neural effects imply that early intervention with electroacupuncture, as in Case no. 1, may have the potential to prevent further deterioration. Even in later stages, as in case no. 2, its impact is significant. Our earlier work has demonstrated that neuroprotection with electroacupuncture results in neurogenesis (56). These observations provide mechanistic insight for the nerve restoration and support the therapeutic role of electroacupuncture. Ultimately, we underscore the patient-centered rationale for the various start times. Our aim was to not only alleviate physical symptoms but also enhance quality of life and improve mood. LARS symptoms, including frequent bowel movements, fecal incontinence, and urgency, can severely impact quality of life. Patient’s urgent requirement for relief prompted the early electroacupuncture therapy in case no. 1. By intervening at 3 months after surgery, we facilitated the spontaneous recuperation of bowel function and alleviated physical discomfort earlier, possibly preventing further deterioration in mental health and daily functioning (9). Clinical research emphasizes the importance of intervention during early period when symptoms are severe, and patients are distressed (57). Numerous therapeutic methods, such as transanal irrigation and early drug interventions, are recommended to begin around 3–12 months after surgery to optimally manage LARS symptoms and help patients return to a normal daily routine (58, 59). Early electroacupuncture intervention can significantly reduce the severity of symptoms and improve psychological health (60). This aligns with the results of the EORTC QLQ-C30 scale in our case reports. On the other hand, the decision of the patient in case no. 2 to pursue electroacupuncture suggests that even after 12 months, persistent LARS symptoms continued to affect daily life, prompting the search for additional therapy. From a clinical perspective, therapy should be provided when the patient needs it most. In summary, despite the different timings of intervention, both patients benefited from electroacupuncture, which reduces bowel dysfunction and improves overall quality of life. This suggests the flexibility and efficacy of electroacupuncture at different stages following rectal cancer surgery. Early electroacupuncture may relieve LARS symptoms and avoid a deterioration in quality of life, while late electroacupuncture could still produce enhancements beyond the spontaneous improvement phase. Electroacupuncture demonstrates potential in the treatment of LARS at different postoperative phases, additional research is required to determine the most effective period for its application in LARS management.

In both cases, the patients’ LARSS scores improved from severe (30–42) to no LARS (0–20), suggesting complete remission of symptoms such as frequent defecation, urgency, and incomplete evacuation. The Wexner scores were significantly reduced in both cases, reflecting an improvement in bowel control. Since the changes in bowel function following rectal cancer surgery have unpredictable negative impacts on patients’ daily lives, emotions, and spirits, resulting in a decline in quality of life (33). Thus, managing LARS should not only focus on the intestinal symptoms but also address the broader effects on quality of life. Accordingly, this study further investigated the EORTC QLQ-C30 scale. Results showed that patients’ EORTC QLQ-C30 functional and overall health status subscale scores increased significantly after a 6-month electroacupuncture treatment targeting Baliao points. This indicates enhancements in physical strength, emotional status, social interaction, and overall health status. Symptom relief was most prominent in fatigue, pain, and diarrhea, suggesting that acupuncture therapy may enhance the quality of life in LARS patients by improving bowel symptoms, which is beneficial for both physical and psychological health. Long-term follow-up results suggest that electroacupuncture may have a sustained effect on improving LARS symptoms. After 6 months of treatment, both patients maintained stable bowel function. LARS-related symptoms did not recur during the follow-up period, and quality of life remained largely unaffected. The follow-up revealed the initial effects of electroacupuncture treatment. The lack of a control group in these case reports generated potential bias. Moreover, the absence of blinding in the evaluation process may affect the outcomes. In future randomized controlled trials, we should incorporate sham acupuncture as a comparator and utilize independent blinded assessors for outcome evaluations. Additionally, extended follow-up periods are necessary to confirm the sustained efficacy of electroacupuncture in the management of LARS.

## 5 Conclusion

These two case reports highlight the potential of electroacupuncture as adjuvant therapy in the management of LARS symptoms following low anterior resection for rectal cancer. As a non-invasive therapy, electroacupuncture has demonstrated potential efficacy in relieving severe LARS symptoms, improving bowel function, and enhancing patients’ quality of life. Electroacupuncture may be a valuable adjuvant treatment option for LARS, but future randomized controlled trials with rigorous design are needed to validate its long-term efficacy.

## Data Availability

The original contributions presented in the study are included in the article/Supplementary material, further inquiries can be directed to the corresponding authors.
